# Navigating technological innovation and rising costs: Assessing economic performance in Chinese manufacturing

**DOI:** 10.1371/journal.pone.0316556

**Published:** 2025-01-03

**Authors:** Songling Wu, Yanqi Si, Xiaoxiang Wang

**Affiliations:** 1 Business College, Henan University of Science and Technology, Luoyang, China; 2 College of Economics and Management, Wenzhou University of Technology, Wenzhou, China; South China Normal University School of Economics and Management, CHINA

## Abstract

Economic performance is an important indicator of the efficiency and quality of a company’s production, which is closely related to the profitability of the company and is crucial for the development of the manufacturing industry.This paper aims to develop a theoretical framework for assessing economic performance within the Chinese manufacturing industry. It achieves this by incorporating inputs, outputs, and energy consumption costs into the production function. By analyzing manufacturing data from 2000 to 2021, it quantifies the impact of various factors, including labor costs and technological advancements, on economic performance. The findings highlight technological progress as the primary driver of economic growth within the Chinese manufacturing sector. Notably, there exists a U-shaped relationship between technical progress and economic performance, suggesting nuanced dynamics at play. Contrary to expectations, the rate of change in per capita wages shows no significant positive impact on economic performance. However, technical progress in the eastern and central regions exhibits a capital bias and positively influences economic performance. Similarly, a U-shaped relationship characterizes the relationship between the western region and manufacturing industry performance. These results underscore the crucial role of technological innovation in sustaining economic performance amid challenges such as rising labor and environmental costs. Emphasizing the reliance on scientific and technological progress emerges as imperative for enhancing the industry’s economic resilience and growth.

## 1. Introduction

In recent years, the Chinese manufacturing industry has faced dual pressures from both developed countries and emerging economies. This has been compounded by the phenomenon of the Chinese labor force reaching the Lewis turning point (Zhang X, Yang J, Wang S,2011) [1]. Additionally, the implementation of re-industrialization strategies in developed nations and the continual elevation of environmental protection standards have contributed to fluctuations in the economic performance of the Chinese manufacturing sector. These fluctuations stem from the rapid and significant decline of its erstwhile low-cost advantage, yet the emergence of a new competitive edge remains elusive (Ji Zhou, Yanhong Zhou et al, 2019) [2]. It’s evident that the developmental trajectory propelled solely by these factors has proven unsustainable. Instead, there’s a growing recognition that innovation-driven growth must serve as the primary impetus behind economic expansion (Shangjin Wei, Zhuan Xie, Xiaobo Zhang, 2017) [3]. Consequently, a critical challenge currently confronting policymakers and industry leaders is how to catalyze technological innovation to bolster the economic performance of the Chinese manufacturing industry amidst escalating labor costs.

Since the inception of economic performance analysis by the Harvard School in 1956, the relationship between factor costs, technological progress, and manufacturing economic performance has been a focal point of economic research. Mansfield (1988) conducted a comparative study of the economic performance of the US manufacturing industry from 1959 to 1977, revealing a declining trend [4]. Similarly, Choudhary S, Nayak R et al (2019) conducted a case study of UK packaging manufacturing SMEs to derive a picture of the current economic performance of small and medium-sized manufacturing firms in the UK [5]. Their research identified factor prices, such as energy, and technological innovations as the primary determinants of economic performance within these companies. Sandrini Luca (2021) argued that while rising costs may escalate production costs and squeeze profit margins, leading to employment pressures and negative impacts on manufacturing enterprises, they can also stimulate enterprise technological innovation, enhance labor productivity, and ignite new sources of economic growth [6].

In the context of the Chinese manufacturing industry, domestic scholars have primarily focused on establishing indicator systems and evaluating directional influences on economic performance. Dai Z, Niu Y et al (2022) [7] and Sun Y, Li L et al (2020) [8] investigated the factors affecting the economic performance of the Chinese manufacturing industry, identifying foreign trade, technological inputs, resources, and labor costs as primary determinants of industry performance. Deng Chuyao and Liu Yang (2022) [9] observed fluctuations in the economic performance of the Chinese manufacturing industry due to continuous rises in labor costs, which undermine the factor advantage, necessitating technological innovation and the reshaping of competitive advantages. Li G, Li X and Wang N(2022) [10] developed an evaluation index system for manufacturing development, highlighting technological innovation as the primary driver behind the high-quality development of the Chinese manufacturing economy, once detached from factor-driven growth. Additionally, Angus C. Chu and Haichao Fan (2021) [11] conducted comparative research on the disparities in the level of manufacturing development across various regions in China, emphasizing technological progress as the basis for high-quality development, rather than factor-driven growth.

Despite the extensive literature on constructing an index system for evaluating Chinese manufacturing economic performance and conducting factor analyses, several unresolved issues persist. Specifically, there is a lack of understanding regarding the effects of rising factor costs and technological progress on Chinese manufacturing economic performance. This gap in knowledge underscores the need for a deeper exploration into the underlying economic principles at play. As the Chinese economy transitions into a new normal phase, relying solely on input-output analysis to explain fluctuations in manufacturing economic performance becomes inadequate. It is imperative to consider the influence of technological innovation and factor costs in this evolving landscape. Under these new circumstances, the economic performance of the manufacturing industry undergoes significant impacts. It is essential to discern which regions within the Chinese manufacturing sector are most susceptible to these influences.

To address these issues, this paper analyzes three main facets utilizing data from the manufacturing industry across all provinces of China (excluding Tibet) from 2001 to 2021. Firstly, it constructs a theoretical framework of economic performance, incorporating the relationship between production inputs and outputs alongside energy consumption costs. By quantifying the mechanisms of various factors’ effects on economic performance, particularly their impact on output costs, this framework aims to establish a theoretical basis for understanding the influence of each factor. Secondly, it improves on previous indicators for evaluating economic performance and introduces new indicators to assess the interaction of factor costs and technological innovation on the economic performance of manufacturing. This represents a departure from previous methodologies and offers a more comprehensive understanding of the dynamics at play. Thirdly, the paper identifies bottlenecks affecting manufacturing industry performance across different Chinese regions, considering regional heterogeneity. It proposes specific countermeasures and recommendations tailored to address these regional disparities and improve the overall performance of the industry in response to the different rates of development of the manufacturing industries in the east, central, and and west of the country.

## 2. Theoretical model

This paper employs the Cobb-Douglas production function as proposed by Teuku Yuri M Zagloel and Rahmat Nurcahyo (2018) [12] and Antonio Ciccone (2002) [13] to analyze industrial productivity. It aims to understand the factors driving improvements in economic performance within the manufacturing sector and their relationship to economic creativity. In this analysis, carbon dioxide emissions, research and development (R&D) investment, and manufacturing value added are integrated into the production function alongside traditional labor and capital input factors. Consequently, the production function for a country’s manufacturing sector can be expressed as *y*_*i*_ = *f*(*l*_*i*_,*k*_*i*_,*e*_*i*_,*Q*_*i*_,*A*_*i*_,Ω_*i*_). Here, *y* the total output of production activities in the manufacturing industry during the period *i*. *l* represents the total number of employees in the manufacturing industry during the period *i*. *k* represents the total physical capital used in the manufacturing industry during the period *i*. Ω represents the efficiency of the economic output of the manufacturing industry during the period *i*. *e* represents the R&D inputs in the development of the manufacturing industry during the period *i*. *Q* represents the value-added in the manufacturing industry during the period *i*. *A* represents the total carbon dioxide emissions in the manufacturing industry during the period *i*. The total output production function for the manufacturing industry is expressed by Eq (1).

$$Y=\Omega {\left(L^{\beta }K^{1-\beta -\gamma }E^{\gamma }\right)}^{\alpha }{\left(\frac{Q}{A}\right)}^{\frac{\lambda -1}{\lambda }}$$ (1)

where *β* and *γ* denote the output elasticity of labor and technology, respectively (0≤*α*≤1,0≤*β*≤1).To derive the output per unit of labor input (Eq 2), both sides of Eq (1) are divided by *L*:

$$\frac{Y}{L}=\Omega {\left[{\left(\frac{K}{L}\right)}^{1-\beta -\gamma }\right]}^{\alpha }E^{\gamma \alpha }Q^{\frac{\lambda -1}{\lambda }}A^{-\frac{\lambda -1}{\lambda }}L^{\alpha -\alpha \gamma -1}$$ (2)

Based on the principle that the marginal output of labor and capital factors of production in the production process is equal to their prices, we can derive:

$$MP_{K}=\Omega L^{\beta \alpha }\left[(1-\beta -\gamma )\alpha \right]K^{(1-\beta -\gamma )\alpha }K^{-1}E^{\gamma \alpha }{\left(\frac{Q}{A}\right)}^{\frac{\lambda -1}{\lambda }}=r$$ (3)

$$MP_{L}=\Omega \left(\beta \alpha \right)L^{\beta \alpha }L^{-1}K^{(1-\beta -\gamma )\alpha }E^{\gamma \alpha }{\left(\frac{Q}{A}\right)}^{\frac{\lambda -1}{\lambda }}=\omega$$ (4)

Here, MP_K_ denotes marginal output of capital, MP_L_ denotes marginal output of labor, r denotes the price of capital, and ω denotes the price of labor. Eqs (3) and (4) signify that the marginal output of capital equals the price of capital (interest), and the marginal output of labor equals the price of labor (wages).

By substituting Eqs (3) and (4) into Eq (1), respectively, we can deduce the following equations:

$$K^{(1-\beta -\gamma )\alpha }=Y\Omega^{-1}L^{-\beta \alpha }E^{-\gamma \alpha }{\left(\frac{Q}{A}\right)}^{-\frac{\lambda -1}{\lambda }}$$ (5)

$$L^{\beta \alpha }=Y\Omega^{-1}K^{-[(1-\beta -\gamma )\alpha ]}E^{-\gamma \alpha }{\left(\frac{Q}{A}\right)}^{-\frac{\lambda -1}{\lambda }}$$ (6)

By substituting Eqs (5) and (6) into Eqs (3) and (4), respectively, we can deduce Eq (7):

$$K=\frac{\alpha (1-\beta -\gamma )Y}{r},L=\frac{\alpha \beta Y}{\omega }$$ (7)

Eq (7) simplifies Eqs (3) and (4), succinctly illustrating the relationship between capital and interest, as well as labor and wages. Integrating Eq (7) into Eq (2) allows for the following deduction:

$$\begin{array}{l} \frac{Y}{L}=\Omega^{\frac{1}{1-\alpha +\alpha \gamma }}{\left[\frac{\alpha (1-\beta -\gamma )}{r}\right]}^{\frac{(1-\beta -\gamma )\alpha }{1-\alpha +\alpha \gamma }}{\left(\alpha \beta \right)}^{\frac{\alpha \gamma }{1-\alpha +\alpha \gamma }}{\left(\frac{\omega }{L}\right)}^{-\frac{\alpha \beta }{1-\alpha +\alpha \gamma }}E^{\frac{\gamma \alpha }{1-\alpha +\alpha \gamma }}Q^{\frac{\frac{\lambda -1}{\lambda }}{1-\alpha +\alpha \gamma }}L^{-\frac{\alpha \beta +\alpha \gamma -\alpha +5}{1-\alpha +\alpha \gamma }}A^{-\frac{\frac{\lambda -1}{\lambda }}{1-\alpha +\alpha \gamma }}\\ =\Omega^{\nu }\Lambda_{r}{\left(\frac{\omega }{L}\right)}^{-\theta }E^{-\eta }Q^{-\delta }L^{-\phi }A^{\mu } \end{array}$$ (8)

Here,$\nu =\frac{1}{1-\alpha +\alpha \gamma }$, $\Lambda_{r}={\left[\frac{\alpha (1-\beta -\gamma )}{r}\right]}^{\frac{(1-\beta -\gamma )\alpha }{1-\alpha +\alpha \gamma }}{\left(\alpha \beta \right)}^{\frac{\alpha \beta }{1-\alpha +\alpha \gamma }}$, $\theta =\frac{\alpha \beta }{1-\alpha +\alpha \gamma }$, $\eta =-\frac{\gamma \alpha }{1-\alpha +\alpha \gamma }$, $\delta =-\frac{\frac{\lambda -1}{\lambda }}{1-\alpha +\alpha \gamma }$, $\varphi =\frac{\alpha \beta +\alpha \gamma -\alpha +5}{1-\alpha +\alpha \gamma }$, $\mu =-\frac{\frac{\lambda -1}{\lambda }}{1-\alpha +\alpha \gamma }$. Where *ν*, Λ_*r*_, *θ*, *η*, *δ*, *ϕ*, and *μ*are all constants. The absolute value is added to *δ* and *μ* to account for their practical significance. The simplification of Eq (8) results in:

$$\Omega ={\Lambda_{r}}^{-\frac{1}{\nu }}{\left(\frac{\omega }{L}\right)}^{\frac{\theta }{\nu }}E^{\frac{\eta }{\nu }}Q^{\frac{\delta }{\nu }}L^{\frac{\phi }{\nu }}A^{-\frac{\mu }{\nu }}{\left(\frac{Y}{L}\right)}^{\frac{1}{\nu }}$$ (9)

Here, $\frac{\omega }{L}$ and *E* are the dependent variables, which represent labor factor costs and R&D inputs essential for technological innovation, respectively.

## 3. Empirical model and variables

### 3.1 Research hypothesis

Chinese manufacturing heavily relies on the resource advantage of cheap labor to maintain its position as one of the major manufacturing countries. Changes in the quantity and cost of labor significantly impact the economic performance of Chinese manufacturing. Rising factor costs, particularly concerning low-skilled labor, affect the industry’s economic performance. As stated by Fan H, Hu Y, Tang L (2021) [14], fluctuations in the quantity and cost of low-skilled labor play a pivotal role in this regard. When the costs of medium- and low-skilled labor escalate, enterprise operating costs rise accordingly, as highlighted by Acemoglu D and Restrepo P (2020) [15]. Xiao Yao and Ma Dandan (2019) observe a shift in the labor market dynamics, with shortages at the lower end becoming prevalent, marking the end of the ’labor surplus’ era [16]. Cai Fang (2023) [17] employs the Lewis binary economic model to elucidate excessive wage increases in China, suggesting the attainment of the ’Lewis inflection point.’ Additionally, Fei Wang and Junjie Xia (2020) underscore the challenge faced by the Chinese manufacturing industry due to the waning demographic dividend and escalating factor prices, leading to a ’double squeeze’ situation [18]. Furthermore, Ma D, Cheng Y et al (2020) emphasizes the diminishing labor cost advantage of Chinese manufacturing compared to countries like Mexico, Colombia, and Russia, with unit labor costs surpassing those of neighboring nations [19]. Overall, the demand for low-cost labor remains consistent across different developmental stages of manufacturing industries. Consequently, this paper proposes the hypothesis:

*H1*: *The economic performance of the manufacturing industry deteriorates with an increase in the rate of change in per capita wages*.

The economic performance of the manufacturing industry is profoundly influenced by its level of technological innovation. Research and development (R&D) investment enhances the adoption of advanced technology, fostering long-term technological advantages and ultimately bolstering economic performance, as argued by Jaime Gómez and Pilar Vargas (2012) and Rabiei and Dadkhah (2014) [20,21]. Scholars often utilize R&D investment as a proxy variable to examine the nexus between technological innovation and enterprise productivity or economic performance, as noted by Thomas A, Rowlands H et al(2016) [22]. Griliches (1985) found a positive impact of technological progress on the economic performance of US manufacturing enterprises [23]. Similarly, Hazarika Natasha(2021) and Heij CV, Volberda HW et al. (2020) discovered significant positive relationships between R&D expenditures and economic performance in their respective studies [24,25]. However, this study suggests a broader perspective that encompasses the promotion of technological progress alongside economic performance improvement in the manufacturing industry. Moreover, it raises the question of evaluating China’s level of technological innovation from a global standpoint, proposing the following hypothesis:

*H2*: *The level of technological innovation correlates positively with the economic performance of the manufacturing industry*.

Considering the 34 provincial-level administrative regions of china, the eastern region, particularly mega-cities like Beijing, Guangzhou, and Shenzhen, has advanced in forming an intelligent manufacturing industry chain. However, smart development in western provinces and cities remains nascent. Disparities in economic development, industrial foundation, technological innovation, and human capital across regions can affect manufacturing economic performance differently. Therefore, this paper suggests the hypothesis:

*H3*: *The interaction between factor cost and technological innovation capacity on manufacturing economic performance varies regionally*.

### 3.2 Empirical model

The econometric model employed in this paper to assess the influence of technological progress on the economic performance of the manufacturing sector in each region is represented by Eq 10. Building upon the preceding analysis, it is plausible that the impact is non-linear. Hence, the squared term of the level of technological progress, along with other control variables, is incorporated into the model.


$$Y=\alpha_{0}+\alpha_{1}X_{1}+\alpha_{2}X_{2}+\alpha_{3}{X_{2}}^{2}+\alpha CV_{S}+r_{year}+r_{province}+\varepsilon$$ (10)

Here, *Y* represents the economic performance of the manufacturing industry, where a higher value indicates better development. *X*_1_ represents the rate of change of per capita wage, while *X*_2_ represents the level of technological progress. It is important to note that the impact of technological progress on economic performance may not be linear, hence the inclusion of the square term of technological progress. Additionally, this paper considers other control variables *CV*_*S*_ that may impact the economic performance of the manufacturing industry. These variables include the growth rate of manufacturing value-added *X*_3_, the rate of employment change *X*_4_, the rate of carbon dioxide emissions change *X*_5_, the growth rate of per capita output *X*_6_, the index of demonstrated comparative advantage of Chinese manufacturing industry *X*_7_, and the index of trade competitiveness of Chinese manufacturing industry *X*_8_. r_year_ indicates that time is controlled in the regression process, while *r*_*province*_ indicates that region is controlled in the regression process, and *ε* denotes the random error term.

### 3.3 Variables description

1. Economic performance
  In this paper, the total return on assets is selected as the metric to gauge the economic performance of the manufacturing industry. It is calculated using the formula: $ROA_{i,t}=\frac{\left(\pi_{i,t}+r_{i,t}\right)}{VAL_{i,t}}$, where *ROA*_*i*,*t*_ is the return on total assets of the manufacturing industry *i* in the province (city) at the end of the *t* year. *π*_*i*,*t*_ denotes the total profit of the manufacturing industry *i* in the province (city) at the end of the *t* year. *π*_*i*,*t*_ represents the interest expense of the manufacturing industry *i* in the province (city) at the end of the *t* year. *VAL*_*i*,*t*_ is the total assets of the manufacturing industry *i* in the province (city) at the end of the *t* year.
2. Rate of change in per capita wages
  The most direct data reflection of the increase in labor costs is the wages of employees in the Chinese manufacturing industry. To capture this phenomenon, the rate of change of the average non-private wages of manufacturing (urban) industries in each province (city) from 2000 to 2021 is selected as a proxy. Consequently, the empirical interval for per capita wages spans from 2001 to 2021.
3. Technological innovation
  The growth rate of R&D investment, Total factor productivity (TFP) and the rate of change of all-employee labor productivity serve as pivotal indicators for measuring the level of technological progress in the manufacturing industry. While acknowledging that the growth rate of R&D investment contributes to technological advancement to a certain extent, this study recognizes the importance of employing a more comprehensive proxy variable. Technological progress should not only be assessed through vertical comparisons over time but also necessitates horizontal comparisons with other countries or regions. Total Factor Productivity (TFP) is a comprehensive indicator that measures the ratio of all factor inputs to total output in a certain period. In this paper, we focus on the study of R&D input as a factor, and focusing on the Chinese national conditions, the most appropriate indicator to measure the level of technological innovation in China’s manufacturing industry is the total labor productivity. So, we adopt the rate of change of total labor productivity as our proxy variable for technological innovation. It is calculated as $LP_{i,t}=\frac{MIA_{i,t}}{L_{i,t}}$. Here, *LP*_*i*,*t*_ is the total labor productivity of the manufacturing industry in the province (city) at the end of the first year. *MIA*_*i*,*t*_ is the manufacturing value added of the manufacturing industry *i* in the province (city) at the end of the *t* year. *L*_*i*,*t*_ is the average number of all employees in the national industry *i* in the province (city) at the end of the *t* year. By calculating the rate of change of total labor productivity from 2001 to 2021, we aim to capture the level of technological innovation in the manufacturing industry.
4. Control variables
  The per capita output growth rate of the manufacturing industry (*X*_6_) is employed as a metric to gauge the economic development level of the manufacturing industry. Additionally, other control variables include the growth rate of value added of the manufacturing industry (*X*_3_), the rate of change of employment (*X*_4_), the rate of change of carbon dioxide emissions (*X*_5_), the index of demonstrated comparative advantage of the Chinese manufacturing industry (*X*_7_), and the index of trade competitiveness of the Chinese manufacturing industry (*X*_8_).

When considering the impact of various factors on the economic performance of the manufacturing industry, several key variables come into play. Firstly, the growth rate of manufacturing value added is a crucial determinant. Scholars such as Burstein and Cravino (2015) [26] and Johnson and Noguera (2012) [27] highlight the significance of the value added rate as a key indicator for measuring industrial economic performance. LIU et al (2020) and others argue that the manufacturing value added rate is not only beneficial but also crucial for the development of the manufacturing industry [28]. Although the estimation of manufacturing value added may differ slightly from actual data due to data availability constraints, this paper utilizes industry value added to estimate manufacturing value added in each province (city), from which the growth rate of manufacturing value added is calculated from 2001 to 2021. Secondly, the rate of change of employment volume plays a significant role. Research by Donghui Shi and Ang Yang (2023) emphasizes the importance of this factor in influencing the economic performance of the manufacturing industry [29]. Given that labor-intensive industries still dominate Chinese manufacturing, where higher employment volumes often correlate with increased profitability for labor-intensive enterprises, the rate of change of employment volume is measured by the rate of change of the number of employees in the manufacturing industry from 2001 to 2021.

Thirdly, the international competitiveness of the industry is a vital consideration. This paper utilizes the index of Chinese manufacturing industry’s demonstrated comparative advantage and the index of Chinese manufacturing industry’s trade competitiveness to assess the status of the domestic manufacturing industry globally. Lastly, environmental factors also significantly impact manufacturing industry performance. Carbon emissions from the Chinese manufacturing industry, primarily stemming from fossil fuels, pose environmental challenges. Enhancing energy efficiency and adopting green technologies are advocated as strategies to mitigate carbon emissions and enhance overall manufacturing performance across various ownership types, as suggested by Cheng Zhang et al (2024) [30] and Liang and Liu (2017) [31].This paper estimates the rate of change of carbon dioxide emissions using the IPCC method, based on the energy consumption of different manufacturing industries and their corresponding carbon dioxide emission coefficients, as outlined by Wang and Tang et al (2020) [32].

### 3.4 Research data

The empirical data in this study were primarily sourced from various authoritative publications, including the China Industrial Statistical Yearbook, National Statistical Yearbook, China Science and Technology Statistical Yearbook, China Energy Statistical Yearbook, IPCC National Greenhouse Gas Guidelines, and provincial statistical yearbooks spanning the period from 2000 to 2021. In instances where data were missing, the plug-in method or weighted average method was employed to fill in the gaps. Furthermore, the main sample excludes the Tibet region due to insufficient and severely missing data, rendering measurement impractical. Additionally, to mitigate the influence of extreme values, the explanatory variables and core explanatory variables underwent a 1% shrinking tail treatment. Ultimately, the study obtained 600 samples from 30 provinces (cities) in China spanning the period from 2001 to 2021, as shown in **Table 1**.

**Table 1:** Descriptive statistics of variables (*N* = 600).

Variable	Mean	Std. dev	Min	Max
Economic performance	0.0708	0.0299	0.0088	0.1504
Rate of change in per capita wages	0.1315	0.0805	-0.0080	0.5232
Change rate of total labor productivity	0.0238	0.0368	0.0000	0.2738
Growth rate of manufacturing value added	0.1407	0.1259	-0.1258	0.6251
Change rate of employment	0.0379	0.0647	0.0000	0.4830
Rate of change in carbon dioxide emissions	0.1322	0.1052	-0.1697	0.4048
Growth rate of output per capita	0.0137	0.0858	-0.2482	0.2637
Explicit comparative advantage index	0.1470	0.1526	-0.2475	0.5772
Trade competitiveness index	0.1407	0.1259	-0.1258	0.6251

Note: Based on the results of the descriptive statistics of the variables, collated by the authors.

## 4. Empirical results and analysis

### 4.1 Baseline regression

The benchmark regression analysis examines the relationship between manufacturing economic performance and the rate of change in per capita wages and all-employee labor productivity using ordinary least squares (OLS) estimation, while controlling for two-way fixed effects of year and province. Table 2 presents the results of this analysis.

**Table 2:** Results of baseline regression.

	(1)	(2)	(3)	(4)	(5)	(6)
	Y	Y	Y	Y	Y	Y
X1	0.0031			0.0064		
	(0.2694)			(0.5472)		
X2		0.0163*			0.0028	
		(1.8053)			(0.1830)	
X2^2^			0.0001***			0.0443***
			(4.2485)			(2.8555)
CVs	No	No	No	Yes	Yes	Yes
Year fe	Yes	Yes	Yes	Yes	Yes	Yes
Province fe	Yes	Yes	Yes	Yes	Yes	Yes
*N*	600	600	600	600	600	600
r2_a	0.7040	0.7061	0.7046	0.7373	0.7376	0.7406
F	42.0843	41.5223	45.1009	43.4098	44.3058	43.7813

Note: 1. *, **, and *** indicate significance levels of 10%, 5%, and 1%, respectively; 2. t-values in parentheses, using robust standard errors; 3. Regression results for control variables and constant terms (CVs and _cons) are omitted.

In column (1), the regression does not include control variables, and it indicates that the rate of change in per capita wages does not significantly affect the economic performance of the manufacturing sector. This finding holds even when control variables are added in column (3), suggesting that changes in per capita wages alone may not necessarily lead to improvements in economic performance amid rising labor costs. This underscores the complexity of factors influencing manufacturing economic performance beyond wage adjustments. Columns (2) and (5) focus on the effect of all-employee labor productivity on the economic performance of the manufacturing sector. In column (2), the regression results show that the coefficient on the rate of change in total labor productivity is significant and positive at the 10 percent level, indicating that increasing total labor productivity significantly enhances the economic performance of the manufacturing sector.

From a global perspective, the full labor productivity of a country’s manufacturing sector has a fuller impact on its economic performance, so control variables related to, for example, manufacturing’s explicit comparative advantage and trade competitiveness are added to the benchmark regression. After adding control variables related to the manufacturing sector’s comparative advantage and trade competitiveness, column (6) reveals that both the primary term and the quadratic term of the rate of change in all-employee labor productivity are significantly positive. This suggests a "U" shaped relationship between economic performance and all-employee labor productivity change. Initially, as all-employee labor productivity increases, it adversely affects manufacturing economic performance, likely due to slower technological progress resulting from insufficient R&D investment. However, beyond a certain threshold, further increases in labor productivity lead to improved economic performance. This phenomenon aligns with the findings of Hsieh et al. (2019) [33]. In summary, the benchmark regression highlights the nuanced relationship between labor-related variables, technological progress, and manufacturing economic performance, emphasizing the importance of considering various factors when assessing the drivers of economic performance in the manufacturing sector.

### 4.2 Endogenous analysis

To address potential endogeneity issues arising from reverse causality in some explanatory variables of the econometric model, this paper employs the lagged one-period economic performance of the manufacturing industry as an instrumental variable. As shown in Table 3, the results of the endogeneity test using the instrumental variable method (2SLS) show that the instrumental variables are significantly positive for the core explanatory variables X1, X2 and X2^2^. The F-values of the first stage regressions are all greater than 10, passing the weak instrumental variable test. p-values of the Kleibergen-PaaprkLM values are all less than 0.1, and the instrumental variables pass the unidentifiable test. Remarkably, the signs of the coefficients of the variables remain largely consistent with those in Table 2. This suggests that the main conclusions drawn from the analysis remain robust even after mitigating the endogeneity problem caused by reverse causation. Overall, the adoption of lagged economic performance as an instrumental variable enhances the credibility of the regression results, affirming the stability and reliability of the findings regarding the relationship between explanatory variables and manufacturing economic performance.

**Table 3:** 2SLS instrumental variable method test results.

	(1)
	Y
X1	4.2641*
	(2.5805)
X2	-0.0339***
	(0.0042)
X2^2^	0.1305**
	(0.0646)
CVs	Yes
Year fe	Yes
Province fe	Yes
Observations	570
Number of province	30

### 4.3 Robustness test

To address the diverse developmental phases of the Chinese manufacturing industry from 2001 to the present, this study narrows the sample interval to the period from 2001 to 2010. The regression results in Table 4 demonstrate a significant and positive regression coefficient for the rate of change of per capita wage during this period. Additionally, the squared term of the rate of change of all-employee labor productivity is positive but insignificant. This suggests that during the high-speed development phase of the Chinese manufacturing industry, wage increases stimulate employee motivation, thereby enhancing the economic performance of manufacturing enterprises. Thus, the main conclusions of this paper remain valid even after accounting for the different developmental stages of the Chinese manufacturing industry.

**Table 4:** Sub-sample regression.

	(1)	(2)	(3)
	Y	Y	Y
X1	0.0178*		
	(1.6832)		
X2		0.0360	
		(1.4661)	
X2^2^			0.0190
			(0.8577)
CVs	Yes	Yes	Yes
Year fe	Yes	Yes	Yes
Province fe	Yes	Yes	Yes
*N*	270	270	270
r2_a	0.7906	0.7890	0.7892
F	37.9596	36.7163	35.9067

Furthermore, to bolster the robustness of the findings, sales profit margin is introduced as a control variable in the regression model. Sales profitability, as emphasized by Sam and Mohd Fazli Mohd (2018) [34], is a crucial factor affecting manufacturing industry economic performance. Profit maximization, indicative of enterprise competitiveness, is captured through sales profit margin, where higher values signify better business conditions and stronger competitiveness. The regression results in Table 5, after incorporating this new control variable, are consistent with the estimated coefficients and significance levels of the benchmark regression in Table 2. This underscores the reliability and consistency of the conclusions drawn in this paper.

**Table 5:** Adding control variables.

	(1)	(2)	(3)
	Y	Y	Y
X1	0.0075		
	(0.8449)		
X2		-0.0065	
		(-0.5430)	
X2^2^			0.0348***
			(2.8491)
CVs	Yes	Yes	Yes
Year fe	Yes	Yes	Yes
Province fe	Yes	Yes	Yes
*N*	600	600	600
r2_a	0.8044	0.8045	0.8063
F	60.9293	62.0076	61.0141

Moreover, to further fortify the robustness of the conclusions, the total return on assets from preceding periods is utilized as an indicator to measure manufacturing industry economic performance. The benchmark regression results in Table 6 reveal that the estimated coefficient value of the secondary indicator of the rate of change of all-employee labor productivity remains significantly positive, indicating its core contribution to enhancing manufacturing industry economic performance. However, the coefficient of the rate of change of per capita wage is not significant, aligning with the findings in Table 2. This suggests that the conclusions of this paper remain intact even with the alternative measurement method for explanatory variables. In summary, the robustness tests conducted through the aforementioned three methods confirm the stability and reliability of the benchmark regression results of this paper, reinforcing the validity of the conclusions drawn.

**Table 6:** Replacing explanatory variables.

	(1)	(2)	(3)
	Y_t+1_	Y_t+1_	Y_t+1_
X1	0.0069		
	(0.5281)		
X2		-0.0010	
		(-0.0544)	
X2^2^			0.0336**
			(2.0016)
CVs	Yes	Yes	Yes
Year fe	Yes	Yes	Yes
Province fe	Yes	Yes	Yes
*N*	570	570	570
r2_a	0.7299	0.7302	0.7318
F	40.6042	41.5244	40.9078

### 4.4 Heterogeneity analysis

Table 7 presents the results of group regression analysis focusing on the impact of the rate of change of all-employee labor productivity in the manufacturing sector on its economic performance across Chinese provinces, divided into eastern, central, and western regions. Drawing on data from the National Bureau of Statistics and literature insights, this regional categorization allows for a more nuanced examination of the relationship between all-employee labor productivity and economic performance. The regression results reveal the varying impacts of all-employee labor productivity on economic performance across different regions of China. By disaggregating the analysis, policymakers and researchers gain valuable insights into the unique dynamics and challenges faced by each region concerning enterprise production technology level and economic development. This approach facilitates targeted interventions and policy measures tailored to the specific needs and characteristics of each region, contributing to more effective and equitable regional development strategies.

**Table 7:** Regression results of regional heterogeneity grouping.

	Eastern region			Central region			Western region		
	(1)	(2)	(3)	(4)	(5)	(6)	(7)	(8)	(9)
	Y	Y	Y	Y	Y	Y	Y	Y	Y
X1	0.0119			-0.0155			0.0001		
	(0.5706)			(-0.4344)			(0.0047)		
X2		0.0291			0.0000			0.0536	
		(0.6772)			(.)			(0.8255)	
X2^2^			-0.0513			-0.0064			0.0504*
			(-1.3444)			(-0.0968)			(1.7062)
CVs	Yes	Yes	Yes	Yes	Yes	Yes	Yes	Yes	Yes
Year fe	Yes	Yes	Yes	Yes	Yes	Yes	Yes	Yes	Yes
*N*	220	220	220	160	160	160	220	220	220
r2_a	0.3998	0.4022	0.4021	0.4895	0.4928	0.4890	0.2592	0.2630	0.2693
F	7.8133	8.3733	8.1784	12.9059	13.4163	13.0202	6.9615	7.2674	7.1790

The results in Table 7 shed light on the nuanced relationship between the rate of change of all-employee labor productivity and the economic performance of the manufacturing industry across China’s eastern, central, and western regions. Notably, in the western region, a significant U-shaped relationship is observed, indicating that when the rate of change of all-employee labor productivity is low, it negatively impacts the economic performance of the manufacturing industry. However, beyond a certain threshold, further increases in labor productivity become beneficial for enhancing economic performance. This phenomenon could be attributed to the western development policy, which has propelled improvements in economic growth rate, scientific and technological innovation capacity, and total factor productivity in the region.

In contrast, the rate of change in per capita wages in the western region does not significantly affect the economic performance of the manufacturing sector, aligning with the results of the benchmark regression for all provinces and cities. For the eastern and central regions, the regression coefficients of the rate of change of per capita wages exhibit insignificance or inconsistency with the benchmark regression results in Table 2. Additionally, the coefficients of full labor productivity and economic performance in these regions are negative and insignificant. This could be attributed to the limitations of using full labor productivity to capture changes in capital quality resulting from investments in new machinery and equipment, particularly in regions like the eastern coastal area where reliance on physical capital may be more pronounced.

### 4.5 Heterogeneity analysis is further extended

Moreover, to further verify the robustness of the conclusions, the real impact of technological progress in the manufacturing industry on its economic performance is further analyzed from the perspective of patent quantity and quality. Building on the research of Park Michael et al (2023) [35], this paper examines both the quantity and quality of innovation, including sales revenue of new products, number of patent applications in high-tech industries (manufacturing industries), proportion of invention patent applications, and proportion of new products in the total output value of manufacturing industries. These multiple measures provide a comprehensive understanding of the technological innovation landscape and economic performance of the manufacturing industry across the eastern, central, and western regions. This depth of analysis enhances the credibility and richness of the conclusions drawn in this study. Table 8 shows these technological innovation variables.

**Table 8:** Symbols and measurement of technological innovation variables.

Variable	Variable symbol	Variable symbol
Innovation_number	Innovation_number1	Ln (new product sales revenue)
	Innovation_number2	Ln (number of patent applications)
Innovation_quality	Innovation_quality1	New product sales revenue/gross sales revenue
	Innovation_quality2	Number of patent applications for inventions/total patent applications

The regression results in Table 9 show that the quantity of innovation plays a significant positive role in the economic performance of the manufacturing industry in the Middle East, which can further affirm that technological progress in the Middle East is characterized by capital bias. The regression results presented in Table 9 reveal a significant positive relationship between the quantity of innovation and the economic performance of the manufacturing industry in the Middle East region. This finding provides further support for the notion that technological progress in the Middle East region is characterized by a bias towards capital. By highlighting the importance of innovation quantity in driving economic performance, the results underscore the role of capital-intensive technological advancements in shaping the manufacturing landscape of the Middle East. This suggests that investments in new machinery, equipment, and other capital-intensive technologies have a substantial impact on improving the economic performance of manufacturing enterprises in the region. Overall, the findings from Table 9 reinforce the understanding of the technological dynamics in the Middle East manufacturing sector, emphasizing the significance of capital-biased technological progress in driving economic growth and competitiveness in the region.

**Table 9:** Estimation of the impact of manufacturing economic performance on the number of patents in the east-central region.

	Eastern region		Central region	
	(1)	(2)	(3)	(4)
	Y	Y	Y	Y
Innovation_number1	0.0015**		0.0105***	
	(2.2936)		(3.7488)	
Innovation_number2		0.0014*		0.0122***
		(1.8925)		(4.9518)
CVs	Yes	Yes	Yes	Yes
Year fe	Yes	Yes	Yes	Yes
*N*	209	209	152	152
r2_a	0.4289	0.4228	0.5560	0.5870
F	8.7293	8.7426	14.3196	13.4934

If only the number of patent applications and revenue from sales of new technologies are used to measure the level of manufacturing innovation, the eastern region has long been in the first place in China. However, some studies believe that in some regions these technological innovations are only a quantitative surge, but not followed up in terms of quality, and there are even strategic innovation behaviors such as watering down with design patents, that is, the overall strength of technological innovation has not progressed much, especially the quality of innovation as measured by the quality of innovation in the manufacturing industry is relatively small, so there is a need to re-metric from the aspect of innovation quality relationship with the economic performance of the manufacturing industry in the eastern region. The results in Table 10 show that the quality of innovation does not play a significant role in the technological progress of manufacturing enterprises in the eastern region, probably because of the strong substitutability of patents in China and the lack of complexity, so the quality of patents fails to fundamentally improve, and also does not have a significant pulling effect on the manufacturing industry’s economic performance.

**Table 10:** Estimated impact of manufacturing economic performance on patent quality in the eastern region.

	(1)	(2)
	Y	Y
Innovation_quality1	0.7160	
	(0.6022)	
Innovation_quality2		0.0037
		(0.1473)
CVs	Yes	Yes
Year fe	Yes	Yes
*N*	209	209
r2_a	0.4289	0.4228
F	8.7293	8.7426

The findings from Table 10 suggest that the quality of innovation does not significantly influence the technological progress of manufacturing enterprises in the eastern region of China. This result may be attributed to several factors, including the strong substitutability of patents in China and the lack of complexity in innovation. Firstly, there may exist a high degree of substitutability among patents, which could diminish the perceived value and impact of individual patents on technological progress and economic performance. Additionally, the lack of complexity in innovation processes and outcomes may limit the extent to which the quality of patents can drive significant improvements in technological progress and economic performance.

Moreover, strategic innovation behaviors, such as watering down with design patents, as suggested by Wang Qiong and Wei Yihan (2023) [36], may further dilute the overall strength of technological innovation in the region. This phenomenon could result in a discrepancy between the quantity and quality of innovation, where a quantitative surge in patent applications does not necessarily translate into substantive improvements in innovation quality. Overall, the lack of significance observed in the relationship between innovation quality and economic performance in the eastern region underscores the need for a more nuanced understanding of innovation dynamics and their impact on manufacturing industry outcomes. Further research may explore additional factors contributing to the observed patterns and delve into the specific mechanisms through which innovation quality influences economic performance in the region.

## 5. Conclusions and recommendations

### 5.1 Conclusion

The empirical analysis conducted on manufacturing industry data in China from 2001 to 2021, excluding Tibet and utilizing a balanced panel dataset comprising 30 provinces (districts), reveals several significant findings regarding the relationship between rising factor costs, technological progress, and economic performance of manufacturing enterprises. The 30 provinces (districts) include: Beijing, Tianjin, Hebei, Shanxi, Inner Mongolia, Liaoning, Jilin, Heilongjiang, Shanghai, Jiangsu, Zhejiang, Anhui, Fujian, Jiangxi, Shandong, Henan, Hubei, Hunan, Guangdong, Guangxi, Hainan, Chongqing, Sichuan, Guizhou, Yunnan, Shaanxi, Gansu, Qinghai, Ningxia and Xinjiang.

Firstly, regarding technological innovation and economic performance, the study uncovers a U-shaped pattern between full labor productivity and the economic performance of the manufacturing industry. Initially, as the rate of change of full labor productivity increases, it adversely affects economic performance. However, beyond a certain threshold, further enhancements in technological progress lead to improvements in economic performance. This suggests that while technological progress is evident in manufacturing enterprises, excessive R&D funding initially inhibits the pace of technological advancement. Once technological progress reaches a certain level, the efficiency of full labor productivity enhances the economic performance of the manufacturing industry.

Secondly, the analysis indicates that the rate of change of per capita wage shows a non-significant positive effect on economic performance. This suggests that despite rising labor costs, solely relying on changes in manufacturing wages do not necessarily lead to improvements in economic performance. Furthermore, in terms of regional heterogeneity, the study reveals that in the eastern and central regions, the coefficients of all-employee labor productivity and manufacturing economic performance are negative and insignificant. This finding suggests a capital-biased nature of technological progress in these regions, where full labor productivity fails to effectively capture changes in capital quality resulting from investments in new machinery and equipment.

Lastly, concerning innovation quantity and quality, the study finds that in the Middle East region, innovation quantity significantly contributes to the economic performance of the manufacturing industry, further confirming the capital-biased characteristics of technological progress in this region. However, in the eastern region, the quality of innovation does not significantly impact technological progress and economic performance. This discrepancy could be attributed to the high substitutability and insufficient complexity of patents in China, leading to a lack of fundamental improvements in patent quality and limited influence on economic performance. Overall, these findings underscore the complex dynamics at play in China’s manufacturing industry, emphasizing the importance of understanding regional disparities and leveraging innovation for sustainable economic growth.

### 5.2 Suggestions

Firstly, according to the level of development of the manufacturing industry in different regions, the flexible implementation of policies to protect the economic performance of different manufacturing enterprises. Enterprises in the central and western regions generally have a lower level of technology, to guide the social capital in real time to increase investment in enterprises in the central and western regions, the introduction of relevant preferential policies to give enterprises R & D subsidies to reduce the cost of research and development of enterprises; focus on playing the technological spillover effect of enterprises in the eastern region, to encourage the eastern capital to increase the direct investment in the central and western regions, to give full play to the advantages of the Chinese large market, to enhance the economic performance of enterprises.

Secondly, optimize China’s business environment. With the loss of China’s labor cost advantage, China’s manufacturing industry faces fierce competition from overseas low-cost regions, and the results of this paper show that the rate of change of per capita wage has limited effect on enhancing the economic performance of the manufacturing industry, which implies that the business environment should be optimized comprehensively to attract capital investment, so that internal and external capitals, the state-owned and the private economy, are treated equally, and the complementary advantages of China’s manufacturing industry are fully brought into play to enhance the manufacturing economy. Performance of the manufacturing economy.

Lastly, encourage China’s high-tech enterprises to increase the research and development of advanced technology. The low quality of innovation in China’s manufacturing industry restricts the improvement of economic performance of China’s manufacturing industry, and should guide the R&D of China’s high-tech enterprises to benchmark against the international first-class enterprises, and the preferential policies and innovation funds should be targeted at the real R&D of advanced technologies rather than the transformation of the business mode and the operation of platforms, so as to enhance the quality of innovation in an overall manner.

## Supporting information

S1 Data (XLSX)

S2 Data (XLS)
